# Minimally Invasive Detection of High-Risk Pancreatic Cystic Neoplasms Using a Novel Multiparametric Single-Molecule Biosensor

**DOI:** 10.1016/j.gastha.2025.100790

**Published:** 2025-09-01

**Authors:** Irene Esposito, Lena Haeberle, Oliver Kuss, Fabrizio Torricelli, Eleonora Macchia, Luisa Torsi, SiMBiT Consortium

**Affiliations:** 1 Institute of Pathology, Heinrich-Heine University and University Hospital of Dusseldorf, Dusseldorf, Germany; 2 Institute for Biometrics and Epidemiology, German Diabetes Center, Leibniz Center for Diabetes Research at Heinrich Heine University Dusseldorf, Dusseldorf, Germany; 3 German Center for Diabetes Research (DZD), Partner Dusseldorf, Dusseldorf, Germany; 4 Centre for Health and Society, Faculty of Medicine, Heinrich Heine University Düsseldorf, Dusseldorf, Germany; 5 Department of Information Engineering, University of Brescia, Brescia, Italy; 6 Department of Pharmacy-Pharmaceutical Science and Centre for Colloid and Surface Science, University of Bari Aldo Moro, Bari, Italy; 7 Faculty of Science and Engineering, Åbo Akademi University, Turku, Finland; 8 Department of Chemistry and Centre for Colloid and Surface Science, University of Bari Aldo Moro, Bari, Italy

Early detection of pancreatic cancer represents the only chance for cure of this extraordinarily aggressive disease. This implies the detection and surgical resection of high-risk cystic lesions, that is, intraductal papillary mucinous neoplasms (IPMN) and mucinous cystic neoplasms (MCN) bearing high-grade (HG) dysplasia. Application of multidisciplinary guidelines has improved the detection of high-risk precursors, but the proportion of false negative and false positive cases remains high.^1^ A further improvement in diagnostics has been reached by the introduction of liquid biopsy approaches focusing on the detection of lesion-specific genetic variants in the cyst fluid obtained during endoscopic ultrasound (EUS) investigation.^2^ For example, the PancreaSeq approach, based on a 74-gene DNA/RNA next generation sequencing panel, reports 82% sensitivity (95% confidence interval [CI]: 0.61-0.93) and 100% specificity (95% CI: 0.91-1.00) for the detection of HG-dysplasia and/or invasive cancer.^3^ However, their high cost and complexity limit such approaches to highly specialized centers, far away from largely accessible point-of-care (POC) testing.

**Figure 1:**
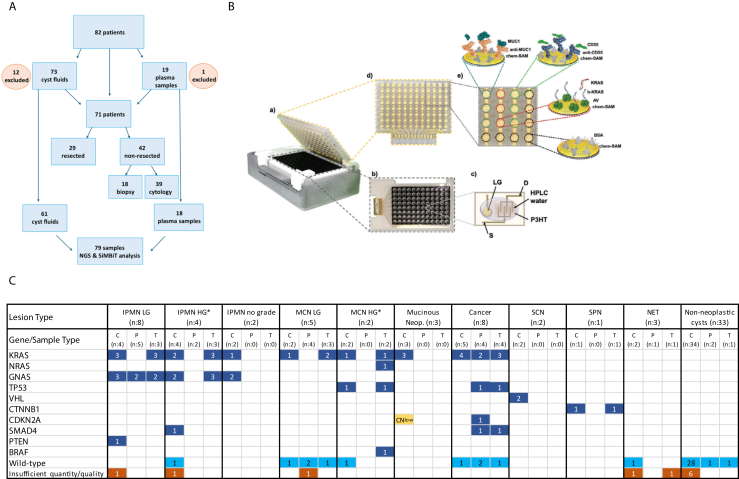
**(A)** Flowchart showing the patients and samples included in the study. **(B)** Illustration of the portable SiMoT ELISA-like 96-sensors bioelectronic array. a) Overview of the device; b) overhead perspective displaying a bottomless ELISA plate glued on top of a plastic foil with the 96 Electrolyte-Gated Organic field effect transistors (EGOFETs) fabricated on it; c) enlarged view of the EGOFET with a later gate (LG) to monitor device stability, and equipped with gold electrodes for the source (S) and drain (D) and coated with a semiconducting polymer (P3HT). HPLC-grade water is used to connect the gates to the channel; d) the three-dimensional (3D) sensing gate is used as a lid; e) enlarged view of a part of the assay showing the biofunctionalized gates in triplicates for the detection of the three parameters MUC1 (cyan), CD55 (green), and *KRAS*^*mut*^ (red). Bovine serum albumin (BSA, black) is used as negative control. **(C)** Oncoprint showing the results of next generation sequencing analysis of the samples analyzed using SiMBiT and included in the suppl. table. Dark blue indicates single nucleotide variants, yellow other alterations, such as reduced gene copy number (CN^low^). Light blue indicates wild-type and orange indicates samples that were excluded due to insufficient volume or quality of the extracted cell-free DNA. The numbers in the boxes indicate the numbers of cases having a given alteration. C, cyst fluid; P, plasma; T, tissue; SCN, serous cystic neoplasm; SPN, solid-pseudopapillary neoplasm; NET, neuroendocrine tumor. ∗Cases with associated cancer were included in the HG category. ELISA, enzyme-linked immunosorbent assay; HPLC, high performance liquid chromatography; KRAS, Kirsten Rat Sarcoma Viral Oncogene Homolog; NRAS, Neuroblastoma RAS Viral Oncogene Homolog; TP53, Tumor Protein p53; VHL, Von Hippel–Lindau tumor suppressor gene; GNAS, Guanine Nucleotide-Binding Protein, Alpha Stimulating; PTEN, Phosphatase and Tensin Homolog; BRAF, v-Raf Murine Sarcoma Viral Oncogene Homolog B; CTNNB1, Catenin Beta 1; CDKN2A, Cyclin-Dependent Kinase Inhibitor 2A; SMAD4, Mothers Against Decapentaplegic Homolog 4. Panel B has been reproduced without changes from: Scandurra C, Björkström K, Caputo M, Sarcina L, Genco E, Modena F, Viola FA, Brunetti C, Kovács-Vajna ZM, Franco CD, Haeberle L, Larizza P, Mancini MT, Österbacka R, Reeves W, Scamarcio G, Wheeler M, Caironi M, Cantatore E, Torricelli F, Esposito I, Macchia E, Torsi L. Analysis of Clinical Samples of Pancreatic Cyst's Lesions with A Multi-Analyte Bioelectronic Simot Array Benchmarked Against Ultrasensitive Chemiluminescent Immunoassay. Adv Sci (Weinh). 2024 Jul;11(27):e2308141. https://doi.org/10.1002/advs.202308141. Epub 2024 Jan 17. PMID: 38234100; PMCID: PMC11251558 according to the terms of https://creativecommons.org/licenses/by/4.0/license, which permits use, distribution and reproduction in any medium, provided the original work is properly cited.

We have previously reported Single Molecule Bio-Electronic Smart System Array for Clinical Testing (SiMBiT), a bioelectronic multiplex array based on single-molecule-with-a-large-transistor (SiMoT) technology.^4^^,^^5^ Briefly, SiMBiT is an ultrasensitive portable diagnostic device that detects biomarkers at the single-molecule level from small fluid samples and delivers results rapidly without complex processing. Here, we report on the results obtained on a series of 92 liquid samples (73 pancreatic cyst fluids and 19 plasma samples) consecutively collected during routine diagnostics from 82 patients (10 with both cyst fluid and plasma) with newly diagnosed cystic lesions of the pancreas (Figure 1A). Clinical guidelines^6^^,^^7^ were applied to determine whether EUS-guided fine-needle aspiration (FNA) or fine-needle biopsy (FNB) was necessary. Cytopathological and/or histopathological evaluation of samples and next generation sequencing-based analysis of cyst fluids was performed at the Institute of Pathology of the University Hospital of Dusseldorf, Germany, a referral center for pancreatobiliary pathology, as described previously.^8^ Diagnosis was reached by integrating clinical, morphological, and sequencing data. Thirty-three patients (40%) underwent surgical resection. The results of cytopathologic/histopathologic (biopsy or resection specimen) analysis were considered the diagnostic gold standard; if no cells/tissue were available, the most probable diagnosis was rendered based on all other parameters. Samples were then analyzed using SiMBiT, focusing on three parameters: *KRAS*^*G12D*^ mutation, the protein MUC1, which is associated with pancreatobiliary IPMN (the most aggressive IPMN subtype) and invasive cancer,^9^ and the protein CD55, a marker of HG-dysplasia in IPMN.^10^ Applying stringent quality criteria, 13 samples (14%; 12 cyst fluids and 1 plasma sample) were excluded from the analysis, which was finally performed on 79 fluids (61 cyst fluids and 18 plasma samples) from 71 patients (34 with non-neoplastic cysts, mostly pseudocysts, and 37 with neoplastic cysts), using the previously described methods (Figure 1B and C, Supplementary Data).^5^ Sensitivities, specificities, positive and negative predictive values (PPV/NPV) were computed 1) for the detection of mucin-producing cysts and 2) the detection of HG lesions (including cancer) in cyst fluids and blood plasma. Statistical analyses were performed using SAS (SAS Institute Inc, Cary, NC, USA), Version 9.4, FREQ procedure.

In the 61 cyst fluids (Figure 1C, Supplementary Data), SiMBiT misclassified only three cases: one low-grade (LG)-IPMN and one LG-MCN were identified as nonmucinous cysts (false negative), and an abscess membrane was classified as LG-mucin-producing neoplasm (false positive). Accordingly, SiMBiT displayed a sensitivity of 91% (95% CI: 71%–99%), a specificity of 99% (95% CI: 87%–100%), a PPV of 95% (95% CI: 76%–100%), and an NPV of 95% (95% CI: 83%–99%) for the detection of mucin-producing neoplasms. Notably, in the plasma samples (n = 18), only one LG-MCN was classified as a nonmucinous cyst (false negative). Therefore, in the plasma analysis, SiMBiT reached a sensitivity of 93% (95% CI: 68%–100%) and a specificity of 100% (95% CI: 37%–100%) for the detection of mucin-producing cystic neoplasms with a PPV of 100% (95% CI: 81%–100%) and an NPV of 75% (95% CI: 19%–99%).

In terms of the clinically even more relevant HG dysplasia detection, SiMBiT correctly identified all HG cases, (11/61 cyst fluids, 18%, and 6/18 plasma samples, 33%). Two cyst fluid cases with molecular but not cytological evidence of a mucin-producing tumor (*KRAS* and/or *GNAS* mutations in the cyst fluid, no cells) were classified by SiMBiT as HG-dysplasia. These patients did not undergo resection and were lost to follow-up, so that a final classification is not possible. Even if we consider these two cases as false positive, SiMBiT reached a sensitivity of 100% (95% CI: 76%–100%) and specificity of 96% (95% CI: 86%–100%) for the detection of HG-mucin-producing neoplasms in cyst fluids (PPV: 85%; 95% CI: 55%–98%; NPV: 100%; 95% CI: 94%–100%). Strikingly, in plasma samples alone, the sensitivity for the detection of HG-mucin-producing neoplasms was 100% (95% CI: 61%–100%), the specificity 100% (95% CI: 78%–100%), the PPV 100% (95% CI: 61%–100%), and the NPV 100% (95% CI: 78%–100%).

These findings show that liquid-based diagnosis of HG-pancreatic cystic neoplasms of the pancreas is feasible at the POC applying the novel SiMoT technology, which has reached the Technology Readiness Level 5 and, by sparing the sequencing, is cost-effective.^5^^,^^11^ Most relevant are the results of diagnostic accuracy obtained using plasma as biologic material: SiMBiT enables highly accurate, noninvasive detection of clinically significant pancreatic cysts directly from blood, eliminating the need for invasive procedures (such as EUS-FNA/FNB) in a significant subset of patients.

Our study has limitations. First, since it is based on a real-world scenario, a selection bias is present due to the selection of patients undergoing EUS-guided FNA/FNB according to clinical decision based on available guidelines on pancreatic cystic neoplasms.^6^^,^^7^ Second, only in 44% of the cyst fluid cases, the diagnostic gold standard, that is, the availability of material for cytologic and/or histologic analysis, was met. This was partly due to the lack of cells in FNA samples, but it also reflects the application of available clinical guidelines for the treatment of pancreatic cystic lesions, which limits the indications for surgical resection.^6^^,^^7^

We envision the use of SiMoT technology at the POC for cyst fluid- and especially blood-based testing in patients with clinically suspicious pancreatic cysts. The clinical decision should always occur in the context of multidisciplinary expertise, considering the still high rates of morbidity and mortality of pancreatic surgery,^12^ which would follow a positive result.
