# Supplementary figures and images for: Minimally Invasive Detection of High-Risk Pancreatic Cystic Neoplasms Using a Novel Multiparametric Single-Molecule Biosensor
Source: Gastro Hep Adv. 2025 Sep 1;4(10):100790. doi: 10.1016/j.gastha.2025.100790 (PMC12547262; doi:10.1016/j.gastha.2025.100790)

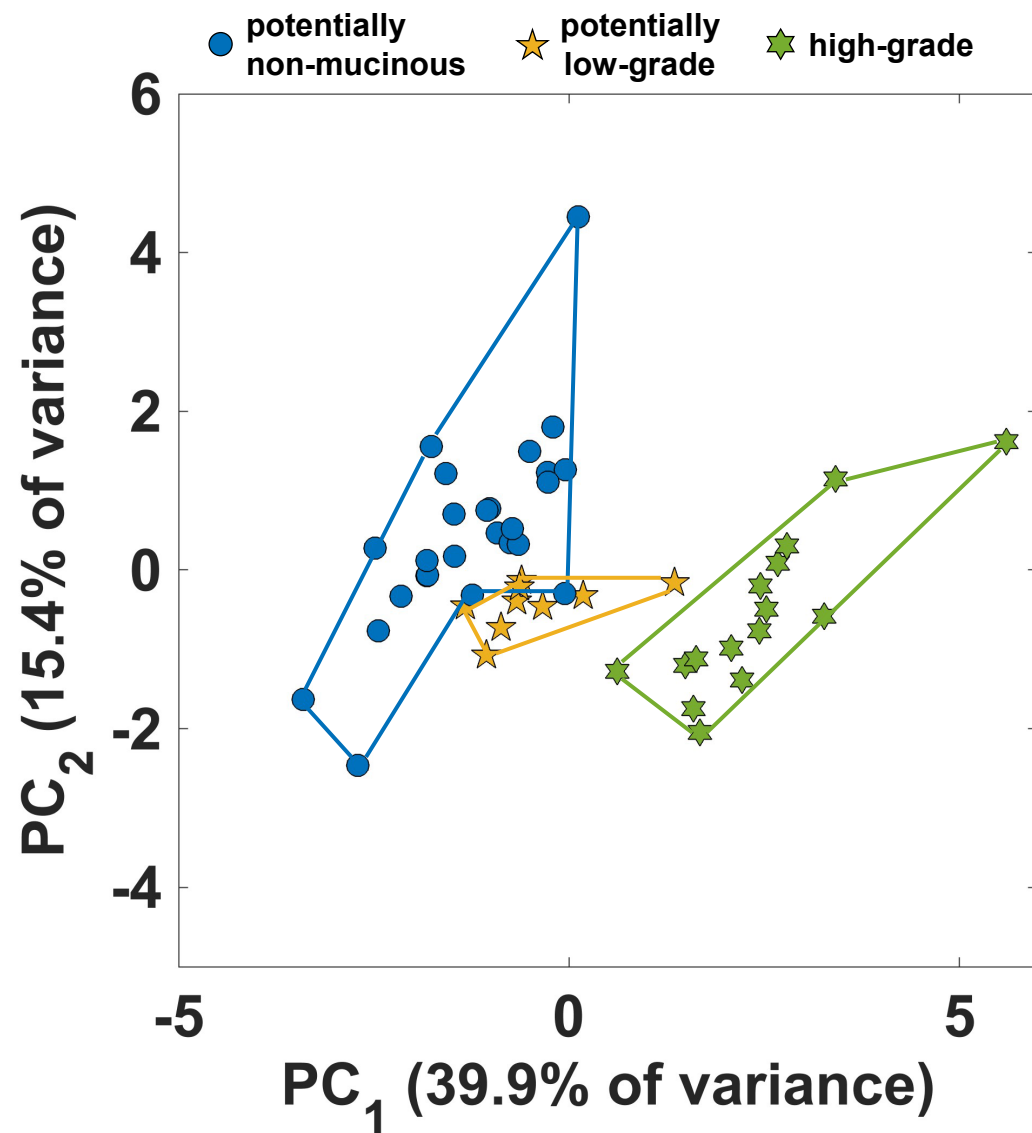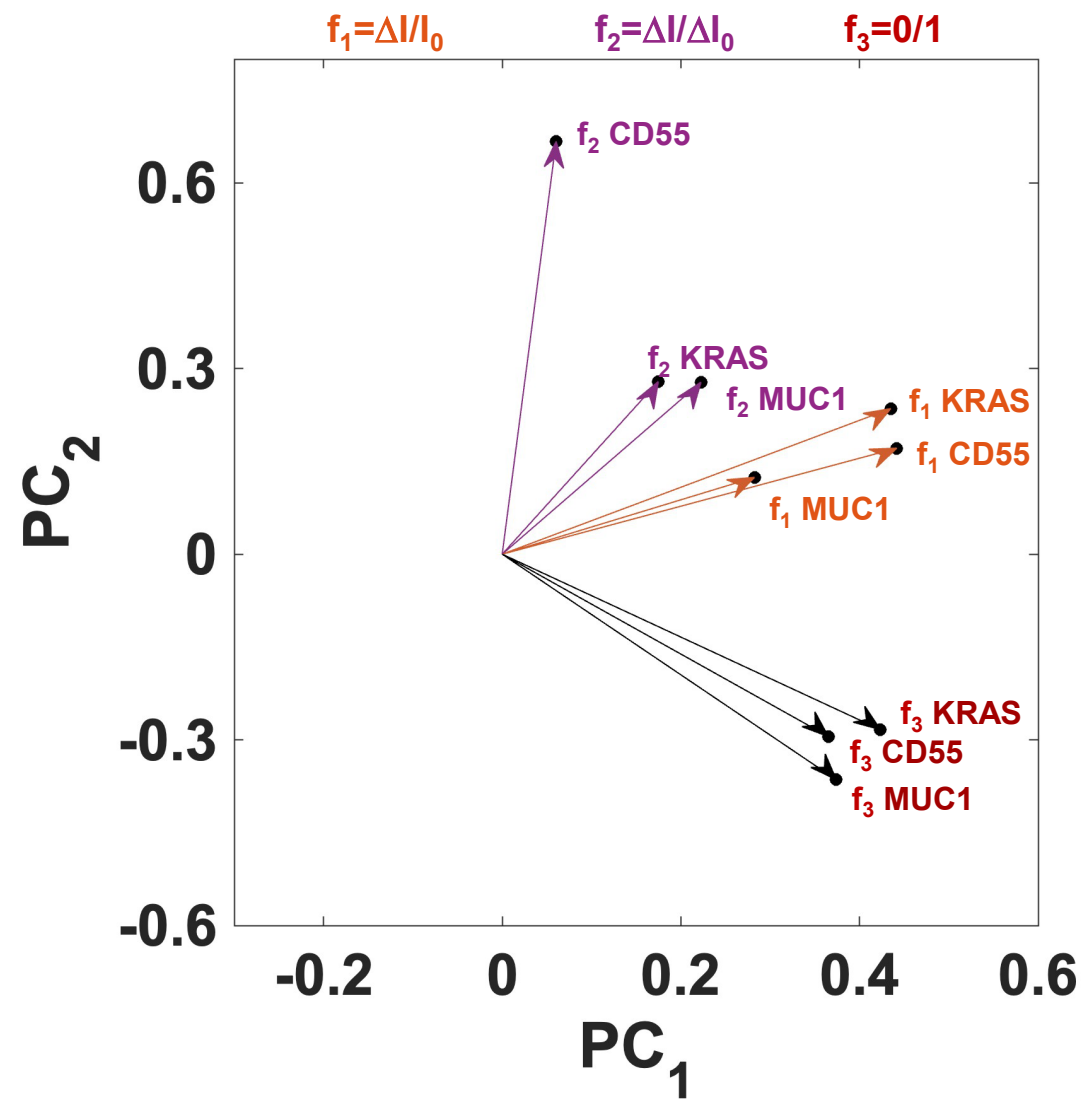

Supplement: Supplementary Figure [file mmc1.pdf]
