# Supplementary material for: Minimally Invasive Detection of High-Risk Pancreatic Cystic Neoplasms Using a Novel Multiparametric Single-Molecule Biosensor
Source: Gastro Hep Adv. 2025 Sep 1;4(10):100790. doi: 10.1016/j.gastha.2025.100790 (PMC12547262; doi:10.1016/j.gastha.2025.100790)
Supplement: Supplementary Data [file mmc2.pdf]

Supplemental Data

<u>Supplemental methods</u>

Patients' enrollment and ethic statement

This study adhered to the principles outlined in the Declaration of Helsinki for medical research involving human participants. Approval for the use of human samples was granted by the local ethics committee of Heinrich Heine University and University Hospital of Dusseldorf, Germany (study numbers: 4664, amendment dated August 9, 2017; 3821, amendment dated June 18, 2019; 22-2170). After completing diagnostic procedures, the collected samples were analyzed retrospectively and stored in a pseudonymized format in compliance with the guidelines established by the ethics committee. Informed consent was obtained for analyses beyond standard diagnostic requirements, such as blood analysis. The study has followed the STARD guideline for the reporting of diagnostic accuracy studies (https://www.equator-network.org/reporting-guidelines/stard/) adapted to the manuscript format.

Sample preparation and NGS analysis of cyst fluids, plasma and tissue samples.

Pancreatic cyst fluids were obtained through routine endoscopic ultrasound-guided fine-needle aspiration or biopsy (EUS-FNA/FNB). Blood samples were collected in Cell-Free DNA BCT® tubes (Streck, Nebraska, USA) following the manufacturer's protocol. Samples were transported promptly to the Institute of Pathology for processing or stored at 4°C if immediate transfer was not feasible. For pancreatic cyst fluids, native samples were centrifuged at 500 × g (1800 rpm) for 5 minutes. Based on pellet volume, up to three smears or cytospins were prepared, fixed with alcohol spray, and stained with Papanicolaou and PAS stains for microscopic examination. Plasma separation from 10 ml whole blood was performed by centrifugation at 600 × g for 20 minutes. Cell-free DNA (cfDNA) was extracted using the QiAamp Circulating Nucleic Acid Kit (Qiagen, Hilden, Germany) and quantified using a custom qPCR assay (Forward primer: 5′-AAACGCCAATCCTGAGTGTC-3′; Reverse primer: 5′-CATAGCTCCTCCGATTCCAT-3′).

Tissue samples were obtained either through FNB or surgical resection. Stained sections were reviewed to select appropriate regions for DNA extraction, which was conducted using the GeneRead DNA FFPE Kit (Qiagen, Hilden, Germany) according to the manufacturer's protocol.

Both cfDNA and tissue-derived DNA were amplified using the Ion AmpliSeq™ Cancer Hotspot Panel v2 and processed using the Ion AmpliSeq™ Library Kit 2.0 with Ion Xpress™ Barcode Adapters. Libraries were quantified with the Ion Library TaqMan™ Quantitation Kit, pooled, and sequenced on an Ion S5™ System using the Ion 520™ & Ion 530™ Kit-OT2.

Primary data analysis was performed on the S5 Torrent Server VM. Variant detection and annotation were conducted using Ion Reporter Software (version 5.12) with standard parameters: minimum allele frequency of 1%, minimum coverage of 500, and a Phred quality score >30. Variants were further analyzed using tools and databases such as Integrative Genomics Viewer (IGV), ClinVar, OncoKB, COSMIC, FATHMM, and PolyPhen-2 to determine their clinical relevance. All reagents and software were provided by Thermo Fisher Scientific (Darmstadt, Germany).

SiMBiT platform

The SiMBiT[13] platform used in this study[5] is based on the single-molecule-with-a-large-transistor (SiMoT) technology.[4] This is a qualitative platform capable of assessing both protein and short DNA sequences directly in serum at a threshold of one marker in a sample of 0.1 ml, with false-positive and false-negative rates falling in the 1 - 5% range.[14] The system has the

footprint of an ELISA plate and features a single-use cartridge with 96 sensors paired with a reusable reader that connects to a computer *via* a USB interface. The reader is operated via a dedicated app run by a standard portable computer.[5]

The biofunctionalization of each gate is a process that involves the deposition of a chemical self-assembled monolayer (chem-SAM) where thiol functional groups covalently bind to the gold layer of the sensing gates, to which specific capturing elements are covalently attached. For MUC1 and CD55 detection, anti-MUC1 and anti-CD55 antibodies are conjugated to the chem-SAM. For $KRAS^{mut}$ detection, avidin is covalently bound to the chem-SAM, while a biotinylated complementary strand of $KRAS^{mut}$ is the probe. Same sensing gates are biofunctionalized with the non-binding bovine serum albumin, serving as negative control to measure the level of the noise. The array of sensing gates is organized into six groups, each containing 16 gates, and each group is assigned to analyze fluids (such as plasma or cysts) from an individual patient. For every patient, the process involves three replicates per biomarker alongside seven negative control tests. The system can process samples from six patients, encompassing three biomarkers and seven negative controls per patient, within a total duration of 90 minutes. A video showing the whole assay can be found at adma202304102-sup-0002-MovieS1.mp4.

SiMBiT multivariate process monitoring

The SiMBiT dataset was analyzed using a machine learning approach to classify samples into high-grade, low-grade mucinous cyst, or non-mucinous categories. A k-Nearest Neighbour (k-NN) classifier was employed, trained on a designated training set, and tested for predictive accuracy using an independent test set, which was intentionally excluded from the training process to avoid overfitting.[5]

Before implementing the classification algorithm, a multivariate quality control approach was employed to validate the results obtained from each SiMBiT analysis. This method generates control charts to monitor the performance of each device of the SiMBiT array, ensuring reliable predictions in operational settings. Faulty devices were identified as statistical outliers by comparing the electronic features acquired during each sensing experiment with those recorded under normal operating conditions (NOC) used to train the algorithm. Specifically, the NOC was established using a SiMBiT training set comprising 50 patient samples (39 cyst fluids and 11 plasma samples). For each patient sample, the relative current shift ($\Delta I/I_0$) measured with the lateral reference gate across all 16 sensors in the array, along with the $\Delta I/I_0$ values from 7 negative control experiments, were extracted, yielding a total of 23 process variables. These 23 features, extracted for each patient, were utilized to compute a Principal Component Analysis (PCA) model, which served as the foundation for constructing the multivariate monitoring chart. The latter has been obtained by the combined analysis of the Hotelling T^2 and Q-residuals control statistics. A violation of the T^2 threshold indicates that the systematic variations are out of control. On the other hand, a violation of the Q threshold indicates that the random noise has significantly changed. Any SiMBiT assay with T^2or Q values exceeding the control limits is flagged as an outlier, indicating it falls outside the model's boundaries. Consequently, such assays are excluded from the classification model. The influence plot, displaying the Q-residuals versus the T^2statistics for the 50 samples in the training set, revealed no outliers, confirming that the training set is a suitable representation of the NOC. Subsequently, the remaining 42 samples, evaluated in a double-blind manner, were projected onto the PCA model derived from the NOC. This analysis identified 13 outliers, which were excluded from further classification. Specifically, one sample exceeded the Hotelling's T^2 threshold, associated with current variations observed in the 7 negative controls. Additionally, 4 samples were classified as outliers based on both the T^2 and Q statistics. Finally, 8 samples were identified as outliers exclusively based on the Q statistic. The Q Contribution plot

indicated that at least four features related to current variations in the Lateral Gate were the dominant contributors to this residual error.

References

13. https://www.simbit-h2020.eu/

14. Macchia E, et al. Adv Mater 2024; 36: 2309705

Suppl. Table 1 – Sample collective and results of the SiMBiT analysis

**Demographic data**	
Age median [range]	63 years [13-84 years]
Female – male	39 (54.92%) – 32 (45.07%)

**Diagnosis**	**N° of pts (%)**	**Biological material**		**SiMBiT**			
		**Cyst fluid**	**Plasma**	**Cyst fluid**		**Plasma**	
**Non-neoplastic cysts**	34 (48)	33	2	33 negative *1 positive*[#]		2 negative	
**IPMN**	13 (18)	11	7				
Low-grade	7	5	5***	4 positive LG	*1 negative*[##]	5 positive LG	
High-grade	2	2	1	2 positive HG		1 positive HG	
High-grade with cancer	2	2	1	2 positive HG		1 positive HG	
No-grade	2	2	0	1 positive LG, *1 positive HG*[$]		-	
**MCN**	7 (10)	4	4				
Low-grade	5	2	4**	1 positive LG	*1 negative*°	3 positive LG	*1 negative*[§]
High-grade	1	1	0	1 positive HG		-	
High-grade with cancer	1	1	0	1 positive HG		-	
**MN**	3 (4)	3	0				
Low-grade	1	1	0	1 positive LG			
No-grade	2	2	0	1 positive LG, *1 positive HG*[$]		-	
**Cancer**	8 (11)	5	4***	5 positive HG		4 positive HG	
**SCN**	2 (3)	2	0	2 negative		-	
**SPN**	1 (2)	1	0	1 negative		-	
**NET**	3 (4)	2	1*	2 negative		1 negative	
**Total**	71	61	18				

False positive and negative cases according to the SiMBiT analysis are in *italic*.

* one case in this category had only plasma samples available for the analysis

** two cases case in this category had only plasma samples available for the analysis

*** three cases in this category had only plasma samples available for the analysis

In this case, a GNAS Q227R mutation was actually found with an extremely low allele frequency (0.26%) and therefore interpreted as negative result at NGS analysis.

this case was classified as IPMN-LG based on the presence of *GNAS* R201H mutation at NGS analysis, whereas *KRAS* was wildtype. However, the SiMBiT panel does not include a mutated *GNAS* sequence.

$ these cases were cautiously considered as false positive for HG dysplasia at statistical analysis, since the degree of dysplasia could not be determined due to the absence of cells in the cytopathology specimens

° *KRAS* wildtype in cyst fluid and tissue

§ case with only plasma sample available

Supplemental figure legend

The figure displays the results of a Principal Component Analysis (PCA) applied to the entire SiMBiT training set. The left panel shows the score plot, which illustrates the distribution of samples based on the first two principal components (PC1 and PC2), while the right panel depicts the loadings of the features contributing to these components. In the left panel, three clusters of patients are clearly distinguishable: high-grade (green hexagrams), potentially low-grade (yellow stars), and potentially non-mucinous (blue circles). The sample distribution demonstrates a marked separation of the high-grade group from the others, predominantly along PC1. The right panel highlights the contributions of individual features to the principal components through the loadings plot. Among these, the feature f1 — representing the variation in current between the baseline and sensing — exhibits the highest loading on PC1 for the protein CD55, surpassing all other features. This finding suggests that the identification of CD55, particularly its f1 feature, plays a pivotal role in capturing the variation associated with PC1 and is strongly linked to the high-grade patient group. These results underscore the significance of CD55 as a key marker for differentiating high-grade patients.
